# The mind-eat program leads to greater improvements in mindful, emotional, and external eating compared to intuitive eating-oriented education in adults with overweight or obesity: a randomized controlled trial

**DOI:** 10.1186/s12966-026-01931-y

**Published:** 2026-05-21

**Authors:** Marion Van Beekum, Angélique Rodhain, Rebecca Shankland, Soumeya Chetouane, Dominique Jullien-Durmont, Camille Le Rouzic, Justine Myzia, Marie Guiraudou, Youadigue Kemba, Catherine Boegner, Jean-Baptiste Bonnet, Vincent Attalin, Abdulkader Jalek, Ariane Sultan, Christophe Leys, Sandrine Péneau, Antoine Avignon

**Affiliations:** 1 Desbrest Institute of Epidemiology and Public Health, IDESP UMR UA11 INSERM, Université Montpellier, Montpellier, France; 2 Nutrition-Diabetes Department, University Hospital of Montpellier, Montpellier, France; 3 Nutritional Epidemiology Research Team (EREN), Epidemiology and Statistics Research Center, Sorbonne Paris Nord University, Inserm, INRAE, Cnam, University of Paris (CRESS), Bobigny, 93017 France; 4 Montpellier Research in Management (MRM), University of Montpellie, Place Eugène Bataillon – CC 19001 – bâtiment 15, Montpellier, 34095 France; 5 Department of Psychology, Univ. Lumière Lyon 2, DIPHE, Bron Cedex, France; 6 Institut Universitaire de France, Paris, France, Paris, France; 7 Faculty of Psychology, Educational Sciences, and Speech and Language Therapy, Université Libre de Bruxelles, Avenue Franklin Roosevelt, 50 - CP191, Brussels, 1050 Belgium

**Keywords:** Mindful eating, Eating behavior, Emotional eating, Intuitive eating, Overweight, Obesity, Randomized controlled trial

## Abstract

**Background:**

Mindful eating interventions target behavioral and emotional processes involved in eating, but most structured programs have been evaluated in English-speaking settings and often compared with passive controls. This randomized controlled trial assessed the effectiveness of the Mind-Eat program, a structured mindfulness-based intervention, versus treatment as usual consisting of a therapeutic patient education (TPE) program grounded in intuitive eating principles, in adults with overweight or obesity.

**Methods:**

In this single-center trial, 66 adults with overweight or obesity were randomized (1:1) after oral agreement. Of these, 56 completed baseline assessment, and 46 with baseline and at least one post-baseline assessment were included in the modified intention-to-treat analysis. The Mind-Eat group completed eight weekly experiential sessions plus one follow-up session, while the comparator group followed the department’s usual TPE pathway. Assessments were conducted at baseline, post-intervention (week 8), and follow-up (week 12). The primary outcome was change in mindful eating, measured with the validated Mind-Eat Scale. Secondary outcomes included disordered and intuitive eating behaviors, psychological well-being, trait mindfulness, physical activity, and weight. Analyses used linear mixed-effects models.

**Results:**

Significant Group × Time interactions favored the Mind-Eat group for mindful eating at week 8 (β = − 0.40, *p* = .002, *d* = − 1.32) and week 12 (β = − 0.31, *p* = .02, *d* = − 1.02). Compared with treatment as usual, Mind-Eat was also associated with greater reductions in emotional eating (and greater improvements in intuitive eating and trait mindfulness. Improvements were observed in both groups across several outcomes. No between-group differences were found for restrained eating, perceived stress, anxiety, depression, physical activity, or weight.

**Conclusions:**

The Mind-Eat program was associated with greater improvements in mindful eating and key eating-related behaviors, particularly emotional and external eating, than an intuitive eating-oriented TPE program delivered as treatment as usual. These findings support the added value of structured mindfulness-based experiential training in obesity care. However, they should be interpreted in light of attrition and the absence of short-term weight effects.

**Trial registration:**

The study protocol is recorded at Clinicaltrials.gov under the number: NCT06157411.

**Supplementary Information:**

The online version contains supplementary material available at 10.1186/s12966-026-01931-y.

## Background

Traditional weight management interventions, which focus on caloric restriction and physical activity, often yield only short-term benefits, with weight regain commonly observed [1]. Behavioral and psychological processes, particularly those related to self-regulation, appear to play an important role in long-term outcomes [2]. Among these, eating behaviors influenced by internal cues and emotional states may be especially relevant. Disordered patterns such as emotional, external, and non-hunger-related eating are prevalent among individuals with overweight or obesity and have been associated with less favorable weight-related outcomes. Alterations in interoceptive processing, including sensitivity to hunger and satiety signals, have been proposed as potential mechanisms underlying these behaviors [3].

Mindfulness-based interventions (MBIs) promote non-judgmental awareness of internal experiences, including bodily sensations such as hunger and satiety, as well as thoughts and emotions. They have shown promise in reducing binge eating and improving self-regulation in people with overweight or obesity [4–7]. Mindful eating (ME), a specific application of MBIs to eating behavior, focuses on restoring an attuned relationship with food through interoceptive and emotional awareness [8, 9]. However, previous ME studies have shown substantial heterogeneity in design. While several relied on passive comparators and lacked standardized, validated tools to assess change [10, 11], others, such as the SHINE trial, used active comparators combining dietary, physical activity, and behavioral components. These differences limit direct comparisons across studies and highlight the need for trials using both active comparators and validated, multidimensional measures of mindful eating [12, 13].

To address these limitations, we developed the Mind-Eat program, a structured, group-based ME intervention tailored to individuals with overweight or obesity. It was adapted from a validated protocol [14] and refined through qualitative feedback from patients in our clinical setting. The program was designed for implementation in real-life care pathways and evaluated using the Mind-Eat Scale, a validated, multidimensional instrument measuring key components of mindful eating [15].

Importantly, we compared the Mind-Eat program to treatment as usual, consisting of a structured therapeutic patient education (TPE) aligned with intuitive eating principles, i.e., reliance on internal cues and non-restrictive eating [16, 17]. While both approaches rely on internal signals, intuitive eating primarily emphasizes cognitive and behavioral regulation of eating, whereas mindful eating focuses on attentional processes and moment-to-moment awareness of eating experiences. This pragmatic comparison allowed us to evaluate the added value of mindfulness-based experiential training beyond standard non-diet, cue-focused education delivered in routine clinical practice.

This randomized controlled trial aimed to evaluate the effectiveness of the Mind-Eat program in improving mindful eating and related behaviors. The primary outcome was the change in mindful eating score, measured using the Mind-Eat Scale. This outcome reflects a proximal behavioral mechanism targeted by the intervention. Secondary outcomes included intuitive and disordered eating behaviors, psychological well-being, physical activity, and weight.

## Methods

### Study design, data collection and ethical considerations

This study was a single-center, two-arm randomized controlled trial (RCT) conducted at the Nutrition-Diabetes Department of Montpellier University Hospital (France). It forms part of the MIND-EAT research project, supported by the French Institute for Public Health Research (IReSP), which aims to develop and evaluate mindful eating tools and interventions.

The study adhered to the principles of the Declaration of Helsinki and received ethical approval from the Institutional Ethics Committee of Bicêtre University Hospital (ID CAAE: 23.03237.000242) on November 14, 2023. It was prospectively registered on ClinicalTrials.gov (NCT06157411). The full study protocol is available upon request. Initial oral consent was obtained from all participants, followed by written informed consent during the first study visit. Study data were collected and managed using Aviitam^®^, a GDPR-compliant and HDS-certified health data platform.

### Participants

Eligible participants were adults aged 18 years or older with a body mass index (BMI) between 25 and 50 kg/m², consulting the nutrition department for the first time for the management of overweight or obesity.

The study was presented to patients during this initial consultation. Individuals who expressed interest were contacted by telephone within 24 to 48 h to confirm participation. Upon oral agreement, participants were randomized using a centralized computer-generated procedure.

Written informed consent was obtained at Visit 1 (V1), prior to baseline assessments and before intervention initiation. Participants who withdrew before V1 did not undergo baseline assessment and were not included in follow-up procedures.

Exclusion criteria at screening included genetic/syndromic obesity, use of weight-affecting medications, prior bariatric surgery, severe psychiatric disorders, pregnancy or breastfeeding, insufficient French fluency, and concurrent study participation.

Post randomization exclusions included becoming pregnant during the study or Initiation of any medication affecting weight.

Both arms included interventions aimed at improving eating regulation through interoceptive awareness, making this study a comparison between two contemporary non-diet approaches.

Minimum attendance thresholds (≥ 3 core workshops for the TPE group and ≥ 5 sessions for the Mind-Eat group) were defined a priori to characterize adherence to the respective intervention protocols, taking into account their structural differences. These thresholds were not used as criteria for inclusion in the primary analysis.

### Randomization and blinding

All outcomes were self-reported through online questionnaires, except for body weight, which was measured by staff blinded to group allocation. Participants were randomized (1:1) to either the mindful eating intervention (Mind-Eat group) or to treatment as usual, consisting of a structured therapeutic patient education (TPE) program aligned with intuitive eating principles.

**Fig. 1:**
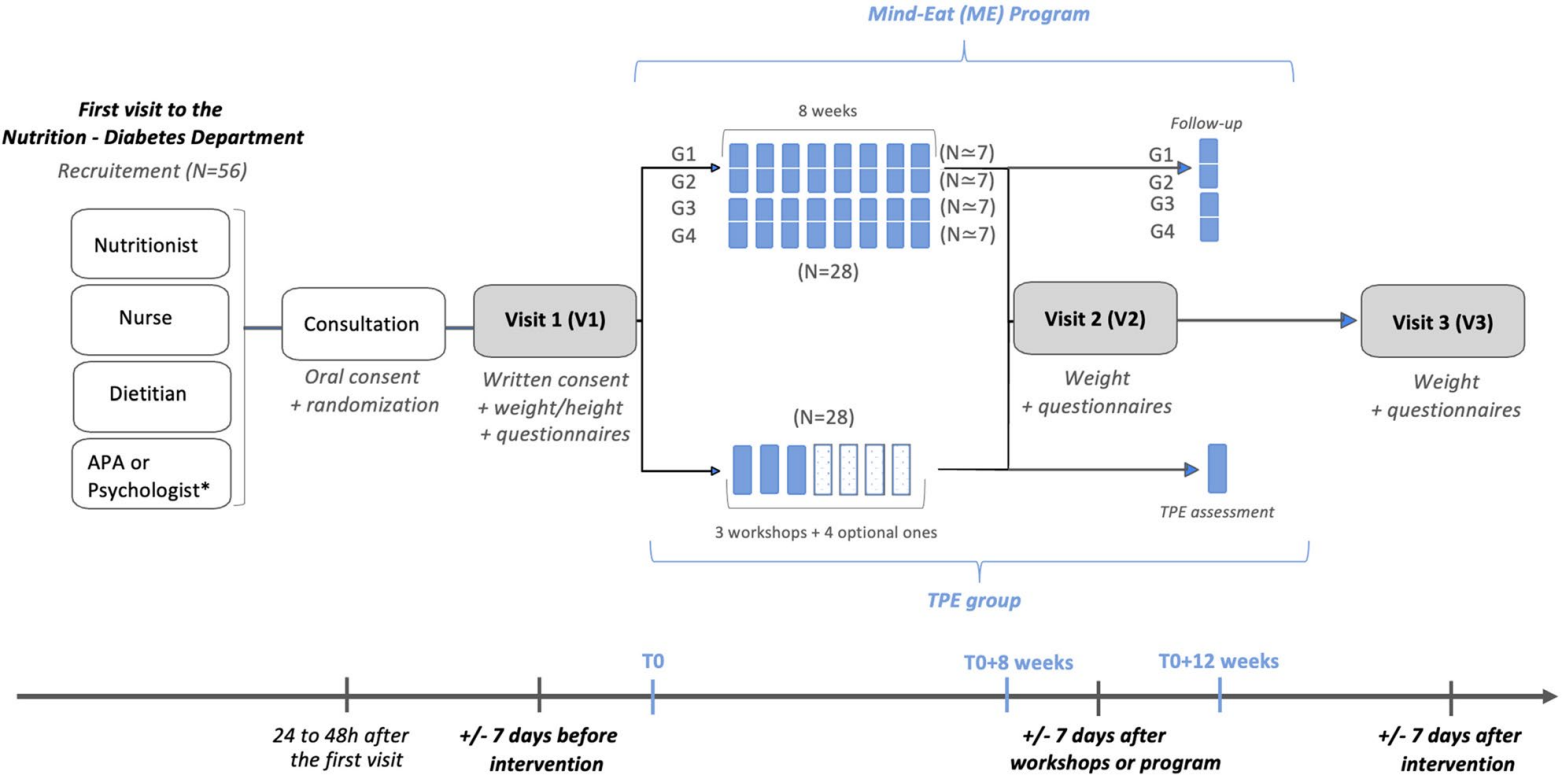
Overview of the study design and baseline assessment procedures. All participants underwent a one-day hospital assessment including four individual consultations (physician, dietitian, therapeutic education nurse, and, depending on individual needs, either a physical activity specialist or a psychologist). *Consultations were tailored according to the patient’s profile.APA: Adapted Physical Activity Professional; G: Group

**Fig. 2:**
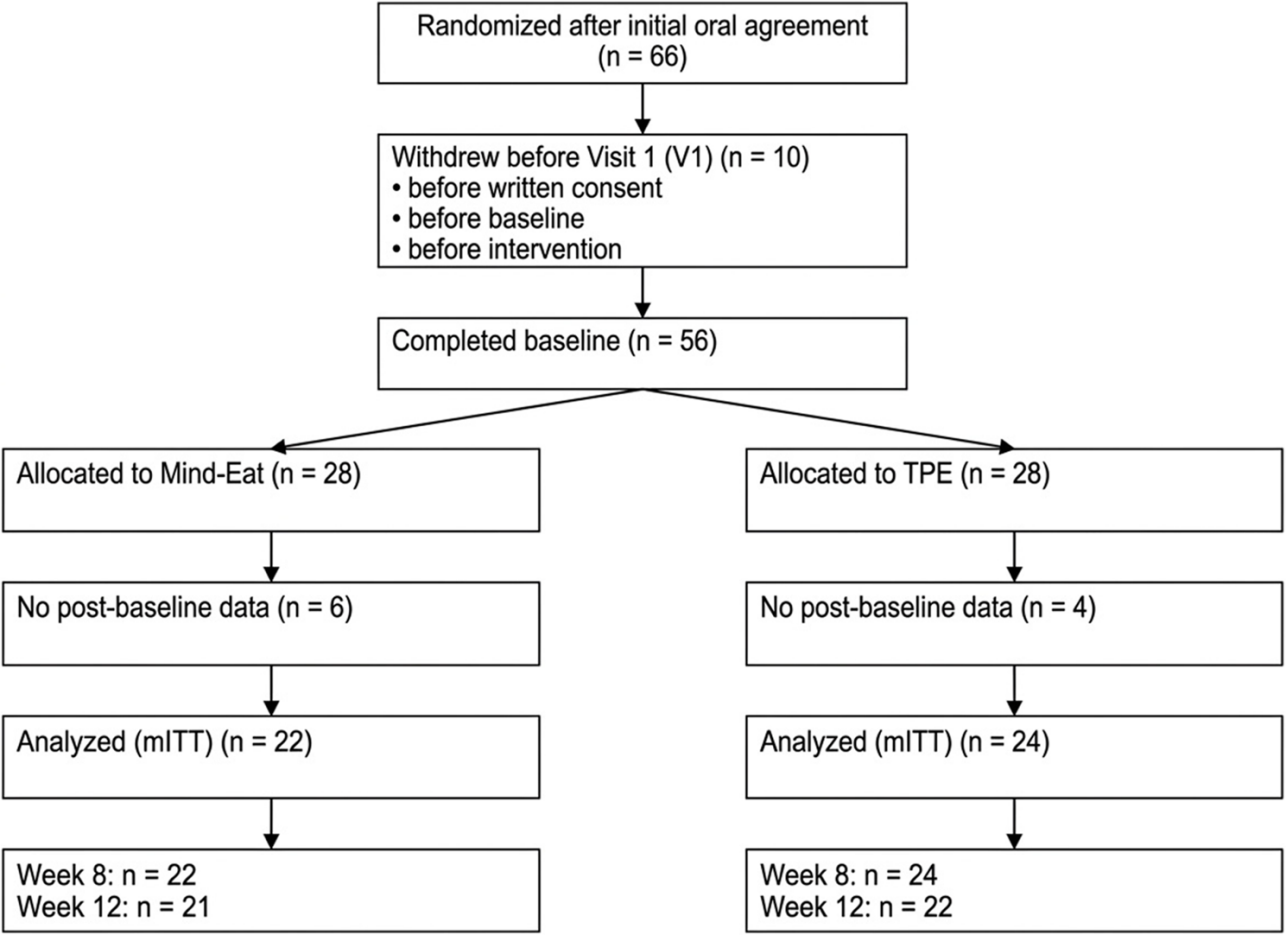
CONSORT flow diagram of participants. Participants were randomized after initial oral agreement. Ten participants withdrew before Visit 1 (V1), that is, before written informed consent, baseline assessment, and intervention initiation. Fifty-six participants completed baseline assessment. The modified intention-to-treat (mITT) population included participants with baseline and at least one post-baseline assessment (Mind-Eat *n* = 22; TPE *n* = 24). Post-intervention assessment was conducted at Week 8, corresponding to the end of the intervention period, and follow-up assessment at Week 12

Randomization was conducted centrally using the ENNOV Clinical platform (CSRandomization module), programmed and overseen by the hospital’s Clinical Research Unit. Allocation occurred at the end of the baseline visit after eligibility confirmation and consent. A schematic overview of the study design is provided in Fig. 1, and participant flow is presented in the CONSORT flow diagram (Fig. 2).

### Interventions

All participants, irrespective of group assignment, underwent a comprehensive baseline assessment conducted during a single day of outpatient hospitalization. This included four individual consultations (approximately 45 min each) with a physician, a dietitian, a therapeutic education nurse, and, depending on individual needs, either a physical activity specialist or a psychologist. All professionals, particularly the dietitian, adopted an approach grounded in intuitive eating, fostering interoceptive awareness and promoting the recognition of internal hunger and satiety cues.

The two interventions differed in format and degree of standardization. The Mind-Eat program followed a fixed 8-week structure with a ninth follow-up session 4 weeks later. Participants were expected to attend at least five of the eight core sessions (Mind-Eat) or a minimum of three core workshops (TPE) to be considered adherent to the protocol, in line with French national guidelines and funding criteria.

### Mind-eat group

The Mind-Eat program is a structured, experiential group intervention developed as part of the MIND-EAT research project. It was adapted from the protocol by Alberts et al. [14] to meet the needs of individuals with overweight or obesity and further refined through qualitative feedback from former participants (Supplemental Data 1). The intervention consisted of eight weekly 90-minute group sessions delivered by a facilitator with dual expertise in mindfulness and clinical nutrition. Each session included (i) formal mindfulness practice (e.g., body scan, mindful breathing), (ii) experiential eating exercises (e.g., mindful eating of a raisin or biscuit), (iii) psychoeducational content on eating behavior, internal cues, and emotional regulation, and (iv) group discussion (Supplemental Data 2). Participants received weekly home practice assignments, including guided audio recordings and a reflective journal. A ninth consolidation session was added one month post-intervention to support long-term integration of skills.

In clinical practice, the program is known as “MangerConscient”, a French-language name chosen to enhance patient engagement and echo public health messaging such as “MangerBouger” from the French National Nutrition and Health Program (PNNS). For clarity, we refer to the program as “Mind-Eat” throughout this manuscript.

### TPE group

Participants in the control arm followed treatment as usual, consisting of the department’s standard therapeutic patient education (TPE) pathway. This included access to a modular set of six thematic workshops (1.5 to 2 h each) covering topics such as hunger and satiety, nutrition, physical activity, sleep, and emotional eating (Supplemental Data 3). The approach was grounded in non-restrictive, internal-cue–driven principles consistent with intuitive eating [16, 17]. One of the workshops, the “Weight Management Follow-up Group”, was held biweekly and could be attended multiple times to support individualized care.

Beyond the minimum of three mandatory workshops, participants were free to attend additional sessions based on their interests and availability. All workshops were open to patients receiving routine care in the department, regardless of study participation, and were delivered by a trained multidisciplinary team including dietitians, nurses, physical activity instructors, and diabetologists. Sessions were conducted either in person or online, following standardized departmental protocols.

A comparative overview of the structure, delivery, and theoretical foundations of the Mind-Eat and TPE programs is provided in Supplemental Data 4.

### Outcome measures

Data were collected at three time points: baseline (Week 0), post-intervention (Week 8), and follow-up (Week 12), i.e., 4 weeks after the intervention concluded.

The primary outcome was change in mindful eating, assessed with the validated 24-item Mind-Eat Scale, developed in a French population [15]. This multidimensional scale includes six subdomains (Awareness, Non-reactivity, Openness, Gratitude, Non-judgement, and Hunger/Satiety), with four items per dimension. Items are rated on a 5-point scale, and total scores range from 1 to 5. The scale has demonstrated good construct validity and internal consistency, as well as test–retest reliability in the validation study.

Secondary outcomes included:

- Disordered eating behaviors: assessed using the Binge Eating Scale (BES) [18] and the Dutch Eating Behavior Questionnaire (DEBQ) [19], which includes subscales for emotional, external, and restrained eating.
- Adaptive eating behavior was assessed using the Intuitive Eating Scale-2 (IES-2) [16], a validated multidimensional instrument comprising three subscales: eating for physical rather than emotional reasons, reliance on hunger and satiety cues, and unconditional permission to eat. Items are rated on a 5-point Likert scale.
- Psychological well-being: measured using the Perceived Stress Scale (PSS) [20] and the Hospital Anxiety and Depression Scale (HAD) [21],
- Trait mindfulness: assessed by the Five Facet Mindfulness Questionnaire (FFMQ) [22],
- Physical activity: assessed via the Ricci-Gagnon scale [23],
- Anthropometric outcome: body weight measured at each visit with a calibrated electronic scale, and height assessed at baseline with a stadiometer.

For all instruments, higher scores indicate greater expression of the measured construct. Detailed descriptions of scale structure, scoring, and psychometric properties are provided in in Supplemental Data 5.

### Sample size calculation

Based on previous studies, we expected a moderate effect size (Cohen’s d = 0.25) for change in mindful eating over time. This assumption was based on the systematic review by Turgon et al. [22], which reported small-to-moderate effects of mindfulness-based interventions on eating-related outcomes [24]. With α = 0.05 and power = 80%, and assuming three repeated measures, the required sample size was estimated at 44 participants using G*Power [25]. To account for potential dropout and to maintain statistical power for secondary outcomes, we increased the planned sample size by 25%, targeting 56 participants.

### Statistical analysis

Baseline sociodemographic and clinical characteristics were summarized using means and standard deviations (SD) for continuous variables, and absolute and relative frequencies for categorical variables.

Longitudinal changes in primary and secondary outcomes were analyzed using linear mixed-effects models (LMM) with a random intercept for each participant to account for intra-individual variability. Fixed effects included time (categorical), group, and their interaction (time × group) to evaluate differential change over time between the Mind-Eat and the TPE groups.

All models were first fitted unadjusted, and then adjusted for age, sex, and baseline BMI. For the primary outcome (Mind-Eat Scale score), additional sensitivity analyses were conducted after adjusting for baseline scores of anxious and depressive symptomatology (HAD-A and HAD-D, subscales of the Hospital Anxiety and Depression Scale) and trait mindfulness (FFMQ), based on previous evidence of their influence on mindful eating behavior [26, 27].

All analyses used a modified intention-to-treat approach, including participants with baseline and at least one post-baseline assessment (*n* = 46). Analyses were conducted using available data without imputation, which may approximate a complete analysis.

A total of 66 participants were randomized following initial oral agreement. Of these, 10 withdrew before Visit 1 (V1), prior to written consent, baseline assessment, and intervention initiation. Among the 56 participants who completed baseline assessment, 10 did not provide any post-baseline data, resulting in a modified intention-to-treat population of 46 participants. The remaining participants (*n* = 46) had varying degrees of missing data at post-intervention (Week 8) or follow-up (Week 12) timepoints.

Missing data were handled using maximum likelihood within the LMM framework, assuming data were missing at random (MAR). This approach utilizes all available data from each participant without requiring imputation, thereby preserving the longitudinal structure of the data while accommodating different patterns of missingness.

Scale scores were computed according to standard scoring procedures using available items, provided that sufficient items were completed, without imputation at the item level.

Minimum attendance thresholds (≥ 3 core workshops for TPE and ≥ 5 sessions for Mind-Eat) were defined a priori to characterize adherence to the intervention protocols, but were not used as criteria for inclusion in the primary analysis.

A sensitivity analysis was also conducted to examine whether changes in mindful eating scores from baseline to follow-up (Week 12) were associated with the number of sessions attended in each group. Spearman correlations were calculated separately for the Mind-Eat and TPE arms.

Given that over 80% of participants attended more than half of the sessions or workshops, protocol adherence was considered satisfactory; no per-protocol analyses were conducted as not deemed necessary.

Given the significant baseline difference in BMI between groups (39.7 ± 4.99 vs. 36.9 ± 4.42 kg/m², *p*<.001), we conducted an additional sensitivity analysis to assess whether this imbalance affected our primary findings. Participants were stratified into BMI tertiles (< 35, 35–40, > 40 kg/m²), and separate linear mixed-effects models were fitted for each tertile to examine Group × Time interactions for the primary outcome (Mind-Eat Scale score).

All statistical tests were two-sided, with significance set at *p* ≤ .05. Analyses were performed using R software (version 4.2.1; R Core Team, 2022).

## Results

### Sample characteristics

A total of 66 participants provided initial oral agreement and were randomized. Of these, 10 withdrew before Visit 1 (V1), before written consent, baseline assessment, and intervention start.

Fifty-six participants completed baseline assessment and entered the study. Among these, 10 did not provide any post-baseline data. Reasons for withdrawal were recorded when available and were mainly related to loss to follow-up after baseline or personal constraints; early withdrawals occurred prior to intervention exposure. The modified intention-to-treat population therefore included 46 participants (22 in the Mind-Eat group and 24 in the TPE group) (Fig. 2).

Groups were comparable at baseline for sex, age, anxiety/depression (HAD), trait mindfulness (FFMQ), and mindful eating scores (Mind-Eat Scale). A difference in baseline BMI was observed between groups and was accounted for in adjusted and sensitivity analyses (Table 1).

**Table 1:** Baseline characteristics of participants in the Mind-Eat (ME) and TPE groups (*N* = 46)

Characteristic	TPE (*n* = 24)	ME (*n* = 22)
Male	7 (29.2)	8 (36.4)
Female	17 (70.8)	14 (63.6)
Age, years	47.5 ± 14.3	49.3 ± 11.3
Weight, kg	112.0 ± 17.9	102.0 ± 17.6
Height, m	1.68 ± 0.10	1.66 ± 0.10
BMI, kg/m²	36.9 ± 4.42	39.7 ± 4.99
Mindfulness (FFMQ total)	130.0 ± 22.6	129.0 ± 18.4
Mindful eating (Mind-Eat Scale), mean ± SD	2.93 ± 0.60	2.83 ± 0.56
Anxiety + Depression (HADS total), mean ± SD	14.3 ± 5.83	14.4 ± 6.01

Values are mean ± SD or *n* (%). No statistical tests were performed in line with current recommendations; apparent baseline differences (e.g., BMI) were considered in sensitivity analyses

Among participants who initiated the intervention, adherence was satisfactory: Mind-Eat participants attended on average 6.9 of 8 sessions (36% attended all), while TPE participants attended 3.9 of 7 workshops (8% attended all). Follow-up attendance was 91% in Mind-Eat and 100% in TPE, with final visit completion rates of 96% and 92%, respectively. No specific session appeared to be consistently under-attended in either group.

### Effects on mindful eating (primary outcome)

**Fig. 3:**
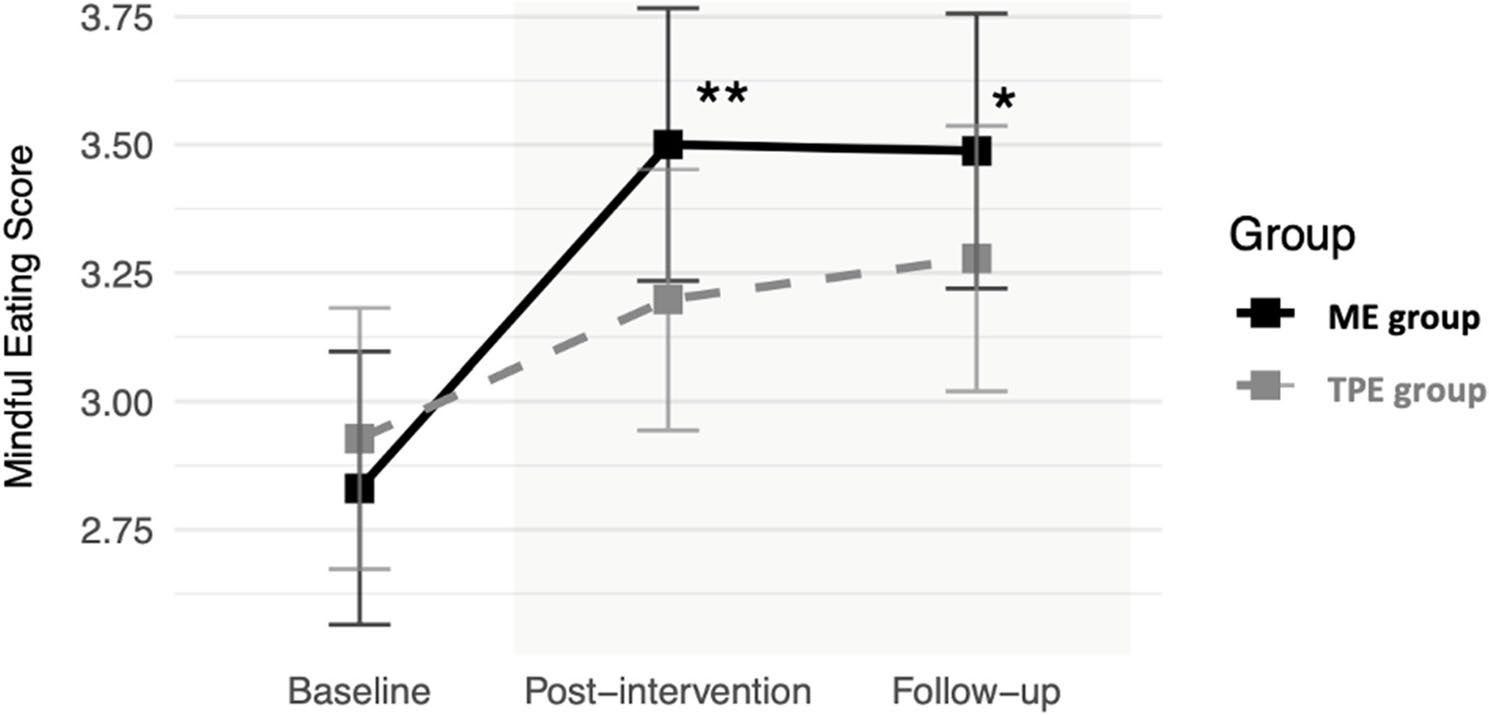
Changes in mindful eating scores over time. Mean (± SD) changes in total Mind-Eat Scale scores from baseline to post-intervention (Week 8) and follow-up (Week 12). *p < .05 (baseline to follow-up); *p < .01 (baseline to post-intervention). Group × Time interaction: *p* = .004 (linear mixed-effects model)

At baseline, mindful eating scores did not differ between groups (β = 0.10, *p* = .60). As shown in Table 2; Fig. 3, mixed-effects models revealed significant Group × Time interactions favoring the Mind-Eat group at post-intervention (β = –0.40, *p* = .002, d = − 1.32) and follow-up (β = –0.31, *p* = .02, d = − 1.02).

**Table 2:** Mind-eat scale total and subdimension scores at baseline, post-intervention, and follow-up: between-group comparisons using mixed-effects models (*N* = 46)

Outcome	TPE (*n* = 24) Mean ± SD	ME (*n* = 22) Mean ± SD	Cohen’s d	β (95% CI)	*p*-value
Mind-Eat Scale total					
- Baseline	2.93 ± 0.60	2.83 ± 0.56	-	-	0.004¹
- Post-intervention	3.20 ± 0.70	3.50 ± 0.60	-1.32	-0.40 (-0.65, -0.15)	0.002
- Follow-up	3.31 ± 0.66	3.50 ± 0.61	-1.02	-0.31 (-0.56, -0.05)	0.020
Non-reactivity					
- Baseline	2.73 ± 0.73	2.52 ± 0.97	-	-	0.013¹
- Post-intervention	2.89 ± 0.83	3.22 ± 0.84	-1.18	-0.54 (-0.92, -0.16)	0.002
- Follow-up	3.14 ± 0.95	3.32 ± 0.82	-0.90	-0.41 (-0.80, -0.02)	0.040
Awareness					
- Baseline	3.15 ± 1.04	3.19 ± 0.88	-	-	0.134¹
- Post-intervention	3.51 ± 0.94	3.99 ± 0.84	-0.84	-0.43 (-0.86, -0.00)	0.050
- Follow-up	3.61 ± 0.83	3.83 ± 0.86	-0.42	-0.22 (-0.65, 0.22)	0.330
Openness					
- Baseline	3.15 ± 0.81	3.73 ± 0.90	-	-	0.625¹
- Post-intervention	3.38 ± 0.97	3.86 ± 0.85	0.23	0.09 (-0.24, 0.43)	0.588
- Follow-up	3.18 ± 0.76	3.85 ± 0.90	-0.19	-0.08 (-0.42, 0.27)	0.664
Gratitude					
- Baseline	2.95 ± 0.87	3.06 ± 0.90	-	-	0.438¹
- Post-intervention	3.33 ± 0.97	3.55 ± 0.98	-0.19	-0.10 (-0.55, 0.34)	0.649
- Follow-up	3.18 ± 0.99	3.62 ± 0.96	-0.54	-0.29 (-0.75, 0.16)	0.207
Non-judgment					
- Baseline	2.88 ± 0.87	2.39 ± 1.12	-	-	0.021¹
- Post-intervention	2.91 ± 1.02	3.03 ± 0.95	-1.09	-0.62 (-1.08, -0.16)	0.010
- Follow-up	3.44 ± 1.10	3.05 ± 1.09	-0.19	-0.11 (-0.58, 0.36)	0.653
Hunger/Satiety					
- Baseline	2.72 ± 1.09	2.09 ± 0.89	-	-	< 0.001¹
- Post-intervention	3.18 ± 1.09	3.35 ± 0.89	-1.44	-0.80 (-1.26, -0.35)	< 0.001
- Follow-up	3.27 ± 1.18	3.31 ± 1.12	-1.31	-0.73 (-1.20, -0.26)	0.003

Values are mean ± SD. Effect sizes (Cohen’s d), β coefficients (95% CI), and *p*-values are derived from linear mixed-effects models including Group × Time interaction

¹ Global Group × Time interaction *p*-values are shown in header rows; contrast *p*-values are presented for post-intervention and follow-up comparisons

Subscale analyses showed greater improvements in Non-reactivity and Hunger/Satiety (Week 8 and 12) and in Non-judgement and Awareness (Week 8 only), with no effects for Openness or Gratitude. No adverse events related to the interventions were reported.

To assess the potential influence of intervention exposure, we conducted a sensitivity analysis examining whether the number of sessions or workshops attended was associated with changes in mindful eating scores (Mind-Eat Scale, Week 12 – Week 0) within each group. In the Mind-Eat group, session attendance correlated positively with improvements in mindful eating (Week 8: *r* = .55, *p* = .008; Week 12: *r* = .47, *p* = .031), indicating a dose–response relationship. No association was observed in the TPE group (Week 8: *r* = –.10, *p* = .63; Week 12: *r* = –.10, *p* = .65). Stratified analyses by BMI tertiles confirmed intervention effectiveness across BMI categories. Results were consistent in sensitivity analyses (see Sensitivity analyses).

### Secondary outcomes

Results from mixed-effects models are shown in Table 3. Intuitive eating (IES-2) improved more in the Mind-Eat group at both post-intervention (β = –0.51, *p* = .002) and follow-up (β = –0.38, *p* = .02). Among subscales, significant effects were found for “Eating for physical rather than emotional reasons” and “Reliance on hunger and satiety cues,”, with no effect for “Unconditional permission to eat.”

**Table 3:** Between-group differences in secondary eating outcomes over time: results from mixed-effects models

Outcome / Time point	TPE (*n* = 24) Mean ± SD	ME (*n* = 22) Mean ± SD	Cohen’s d	β (95% CI)	*p*-value
Intuitive eating (IES-2)					0.004¹
– Baseline	2.97 ± 0.66	2.61 ± 0.74	-	-	
– Post-intervention	3.24 ± 0.68	3.39 ± 0.48	-1.35	-0.51 (-0.82, -0.20)	0.002
– Follow-up	3.41 ± 0.83	3.43 ± 0.55	-1.01	-0.38 (-0.70, -0.07)	0.019
Eating for physical rather than emotional reasons					0.066¹
– Baseline	3.05 ± 0.99	2.63 ± 1.17	-	-	
– Post-intervention	3.30 ± 0.95	3.41 ± 0.81	-0.97	-0.52 (-0.95, -0.08)	0.002
– Follow-up	3.43 ± 0.99	3.34 ± 0.62	-0.59	-0.31 (-0.76, 0.13)	0.170
Reliance on hunger and satiety cues					0.006¹
– Baseline	2.76 ± 0.95	2.21 ± 1.09	-	-	
– Post-intervention	3.21 ± 0.82	3.39 ± 0.95	-1.28	-0.73 (-1.20, -0.26)	0.003
– Follow-up	3.47 ± 1.17	3.43 ± 1.13	-0.99	-0.57 (-1.04, -0.09)	0.022
Unconditional permission to eat					0.517¹
– Baseline	3.12 ± 0.87	3.18 ± 0.93	-	-	
– Post-intervention	3.16 ± 0.78	3.39 ± 0.85	-0.32	-0.17 (-0.61, 0.26)	0.440
– Follow-up	3.28 ± 0.86	3.63 ± 0.77	-0.47	-0.25 (-0.70, 0.19)	0.266
Binge eating (BES)²					0.030¹
– Baseline	13.42 ± 7.88	17.56 ± 8.26	-	-	
– Post-intervention	10.92 ± 7.99	11.77 ± 5.06	0.85	3.27 (0.17, 6.38)	0.044
– Follow-up	9.00 ± 7.16	9.47 ± 6.40	1.06	4.06 (0.88, 7.24)	0.015
Restrained eating (DEBQ)					0.322¹
– Baseline	2.61 ± 0.70	2.80 ± 0.80	-	-	
– Post-intervention	2.70 ± 0.73	2.80 ± 0.88	0.20	0.08 (-0.25, 0.42)	0.631
– Follow-up	2.83 ± 0.84	2.73 ± 0.88	0.63	0.26 (-0.09, 0.61)	0.142
Emotional eating (DEBQ)					0.003¹
– Baseline	2.52 ± 1.18	3.03 ± 1.20	-	-	
– Post-intervention	2.39 ± 1.17	2.24 ± 0.82	1.38	0.66 (0.27, 1.05)	0.001
– Follow-up	2.22 ± 1.12	2.24 ± 0.97	1.02	0.49 (0.09, 0.88)	0.020
External eating (DEBQ)					< 0.001¹
– Baseline	2.82 ± 0.69	3.17 ± 0.68	-	-	
– Post-intervention	2.76 ± 0.61	2.67 ± 0.62	1.48	0.44 (0.19, 0.68)	< 0.001
– Follow-up	2.52 ± 0.66	2.59 ± 0.71	1.16	0.34 (0.09, 0.59)	0.008

Values are mean ± SD. Effect sizes (Cohen’s d), β coefficients (95% CI), and *p*-values are derived from linear mixed-effects models including Group × Time interaction

¹ Global Group × Time interaction *p*-values are shown in header rows; contrast *p*-values are presented for post-intervention and follow-up comparisons

² Multivariate models adjusted for sex, age, and baseline BMI

Binge eating symptoms decreased more in the Mind-Eat group, significant in adjusted models (*p* = .009) but borderline in unadjusted tests (*p* ≈ .06).

Restrained eating (DEBQ) did not differ between groups, while emotional eating (β = 0.66, *p* = .001 at week 8; β = 0.49, *p* = .02 at week 12) and external eating (β = 0.44, *p* < .001 and β = 0.34, *p* = .008) decreased more in the Mind-Eat group.

As shown in Table 4, trait mindfulness (FFMQ) increased significantly more in the Mind-Eat group (β = − 13.81, *p* = .007 at week 8; β = − 12.16, *p* = .01 at week 12). No between-group differences were observed for physical activity (Ricci-Gagnon), anxiety or depression (HAD), perceived stress (PSS) or weight.

**Table 4:** Mixed effects model results for between-group comparisons on secondary psychobehavioral outcomes

Outcome / Time point	TPE (*n* = 24) Mean ± SD	ME (*n* = 22) Mean ± SD	Cohen’s d	β (95% CI)	*p*-value
Physical activity (Ricci-Gagnon)					0.921¹
– Baseline	20.46 ± 8.21	19.09 ± 8.77	-	-	
– Post-intervention	20.46 ± 8.15	19.86 ± 9.20	-0.17	-0.77 (-1.90, 0.68)	0.690
– Follow-up	20.86 ± 8.08	19.71 ± 9.30	-0.06	-0.29 (-0.89, 0.59)	0.886
Anxiety and depression (HADS total)					0.480¹
– Baseline	14.40 ± 6.01	14.30 ± 5.83	-	-	
– Post-intervention	11.50 ± 5.49	10.04 ± 5.45	0.32	1.03 (-1.62, 3.69)	0.447
– Follow-up	11.00 ± 5.78	9.62 ± 6.59	0.51	1.65 (-1.06, 4.37)	0.234
– Anxiety					0.370¹
– Baseline	8.46 ± 4.49	8.32 ± 3.24	-	-	
– Post-intervention	6.83 ± 3.45	6.04 ± 2.70	0.34	0.65 (-0.94, 2.23)	0.418
– Follow-up	6.68 ± 3.14	5.62 ± 3.02	0.60	1.14 (-0.48, 2.76)	0.164
– Depression					0.795¹
– Baseline	5.92 ± 2.59	6.00 ± 3.53	-	-	
– Post-intervention	4.67 ± 2.70	4.36 ± 3.54	0.21	0.39 (-1.13, 1.90)	0.618
– Follow-up	4.36 ± 3.35	4.00 ± 4.10	0.28	0.51 (-1.04, 2.06)	0.522
Stress (PSS)					0.510¹
– Baseline	16.46 ± 8.91	19.73 ± 5.98	-	-	
– Post-intervention	15.20 ± 7.73	16.23 ± 8.86	0.41	2.25 (-2.28, 6.77)	0.326
– Follow-up	14.00 ± 9.58	15.62 ± 9.89	0.43	2.37 (-2.26, 6.99)	0.313
Mindfulness (FFMQ total)²					0.007¹
– Baseline	130.04 ± 22.55	129.46 ± 18.38	-	-	
– Post-intervention	132.92 ± 23.24	146.14 ± 16.39	-1.14	-13.81 (-23.10, -4.52)	0.005
– Follow-up	138.82 ± 23.14	148.71 ± 19.83	-0.84	-12.16 (-21.67, -2.65)	0.014
Weight change (kg)					0.833¹
– Post-intervention vs. Baseline	-0.50 ± 2.18	-0.23 ± 3.38	-	-	
– Follow-up vs. Post-intervention	-0.47 ± 1.94	-0.01 ± 0.95	-0.09	-0.17 (-1.75, 1.41)	0.832
– Follow-up vs. Baseline	-0.95 ± 3.23	-0.18 ± 3.48	-0.26	-0.48 (-2.08, 1.12)	0.552

Values are mean ± SD. Effect sizes (Cohen’s d), β coefficients (95% CI), and *p*-values are derived from linear mixed-effects models including Group × Time interaction

¹ Global Group × Time interaction *p*-values are shown in header rows; contrast *p*-values are presented for post-intervention and follow-up comparisons

² Models adjusted for sex, age, and baseline BMI

### Sensitivity analyses

Sensitivity analyses confirmed the robustness of the findings. Including age, sex, and baseline BMI as covariates did not improve model fit or alter effect significance, supporting the unadjusted model as the most parsimonious. Baseline mindfulness predicted greater change (*p* = .008) ; however, additional adjustments for baseline mindfulness, anxiety, and depression did not alter the direction or significance of the results.

## Discussion

This study evaluated the effects of a structured mindful eating program, delivered in a real-world clinical setting and designed to target core mechanisms such as interoceptive awareness and non-reactivity. Compared to treatment as usual, consisting of a therapeutic patient education (TPE) program grounded in intuitive eating principles, the Mind-Eat intervention was associated with significantly greater improvements in mindful eating behaviors among participants included in the analysis, suggesting that structured experiential training may provide added value beyond cognitive-educational approaches. These effects were observed both immediately after the 8-week intervention and sustained at 12-week follow-up, with large effect sizes particularly in the domains of non-reactivity, non-judgment, and awareness of hunger and satiety cues. To our knowledge, this is one of the first trials to use the Mind-Eat Scale, a recently validated multidimensional instrument developed in French, allowing a detailed assessment of mindful eating through both a total score and specific subdomains. Improvements were observed in both groups across several outcomes, consistent with previous studies of mindful and intuitive eating interventions and reflecting shared underlying principles. However, the greater improvements observed in the Mind-Eat group suggest an additional contribution of structured mindfulness training.

Mindful and intuitive eating scores can be interpreted as proximal mechanisms of change rather than distal clinical outcomes. Their improvement, together with changes in disordered eating and psychological well-being, supports their relevance as meaningful intervention targets.

Our findings should be interpreted in the context of the SHINE study [12, 13], which reported improvements in mindful eating and metabolic outcomes over longer follow-up periods. However, SHINE combined mindfulness with dietary and physical activity components, whereas our intervention specifically targeted eating behaviors without an explicit weight-loss objective. These differences in design and endpoints likely explain the absence of metabolic effects in our study and support the complementary nature of our findings.

Using a comparator reflecting treatment as usual, grounded in intuitive eating represents both a strength and a challenge: this rigorous design sets a high bar for detecting incremental benefits, and the fact that Mind-Eat was associated with greater improvement than TPE suggests that experiential mindfulness training may provide additional therapeutic value.

Beyond mindful eating, both groups showed improvements in several outcomes, consistent with prior studies of mindful and intuitive eating interventions. However, the Mind-Eat group showedgreater improvements in intuitive eating, reductions in emotional and external eating, and increased trait mindfulness, confirming the psychological specificity of the intervention. No between-group differences were observed for binge eating severity (BES), weight, stress, or depressive symptoms, consistent with previous short-term mindfulness trials [5–7]. These findings align with prior research suggesting that mindfulness-based eating interventions can reduce emotional dysregulation and maladaptive eating patterns while enhancing interoceptive awareness [9, 28–30]. The absence of differential effects on stress may reflect the short duration of the intervention and its primary focus on eating behavior rather than broader stress regulation.

Both interventions were grounded in non-restrictive, internal-cue–driven frameworks. While the TPE group followed a flexible, intuitive eating–oriented format, the Mind-Eat program implemented a highly structured experiential curriculum centered on mindfulness practice. This design allowed us to evaluate the specific contribution of formal mindfulness training beyond a contemporary, non-diet nutritional approach.

A notable contribution of this study is the demonstration of a dose–response relationship within the Mind-Eat group, where higher session attendance was associated with greater improvements in mindful eating scores. This association was not observed in the TPE group, likely because this analysis focused on mindful eating, whereas TPE primarily targets intuitive eating–related processes. This suggests that intervention intensity may be particularly relevant in mindfulness-based programs and highlights the importance of accounting for actual participation when interpreting treatment effects.

Although no significant weight change was observed in either group at 12 weeks, this aligns with prior mindfulness-based trials and reflects the program’s behavioral rather than weight-loss focus [6, 7].

Our results further revealed convergence between conceptually related outcomes. Improvements in emotional eating (DEBQ) mirrored those in the IES-2 subscale *Eating for physical rather than emotional reasons*. Likewise, improvements in the Mind-Eat subscale *Awareness of hunger and satiety cues* corresponded with increases in *Reliance on hunger and satiety cues* (IES-2), reinforcing the internal consistency and specificity of the intervention’s effects. In contrast, no significant changes were observed for the *Openness* or *Gratitude* subdomains of the Mind-Eat Scale, two novel dimensions introduced in the scale’s development. This may reflect the fact that these constructs were not explicitly targeted by the intervention and might require either more intensive practice or longer-term follow-up to show measurable effects.

Finally, our findings add to the growing empirical support for the use of the Mind-Eat Scale. Although originally validated in an independent sample [15], this study provides additional evidence of the instrument’s sensitivity to change and convergent validity with established constructs. Nevertheless, the minimal clinically important difference (MCID) for the Mind-Eat Scale has not yet been established, which limits the ability to translate observed effect sizes into clinical significance. Future studies should aim to determine thresholds for clinically meaningful change.

This study has several strengths, including its pragmatic RCT designand the use of a comparator reflecting treatment as usual, consisting of a structured therapeutic patient education (TPE) program rooted in intuitive eating principles. Rather than comparing against inactive controls, this design allowed us to evaluate whether experiential mindfulness training was associated with incremental benefits beyond cognitive-educational approaches, representing a stringent and clinically relevant comparison.

Among participants who initiated the intervention, adherence to the program was satisfactory, with consistent participation across sessions. Despite these similarities, the Mind-Eat intervention was associated with greater improvements across multiple domains compared with TPE, highlighting the potential added clinical value of a structured mindfulness-based experiential program. These findings extend prior work in the field, including the SHINE trial [12, 13], by focusing on behavioral mechanisms within a pragmatic clinical setting.

Despite these encouraging findings, several limitations must be acknowledged. First, the modest sample size was adequate for the primary outcome but may have limited power for secondary outcomes. Second, as multiple secondary outcomes and subscales were tested, some significant findings may reflect chance associations.

Third, Attrition should be considered when interpreting these findings. Notably, 10 participants withdrew before baseline assessment and prior to any exposure to the intervention; therefore, these early withdrawals do not reflect treatment acceptability. Among participants who entered the study, 10 did not provide post-baseline data, which limited their inclusion in the modified intention-to-treat analysis. While mixed-effects models using maximum likelihood estimation allow inclusion of all available data under the missing-at-random assumption, without requiring imputation, the absence of outcome data for these participants may still affect generalizability. Overall, adherence among participants who initiated the intervention was satisfactory, but conclusions should be interpreted in light of this attrition.

Fourth, despite successful randomization, groups differed on baseline BMI (39.7 ± 4.99 vs. 36.9 ± 4.42 kg/m²); stratified analyses by BMI tertiles indicated that effects persisted across BMI categories. Fifth, intervention intensity differed (~ 12 h for Mind-Eat vs. ~ 4–14 h for TPE), which complicates causal attribution, although the dose–response observed only in Mind-Eat argues against a pure exposure-time effect. Sixth, the predominance of female participants, though reflective of typical obesity care populations, limits generalizability to men. Finally, while the absence of short-term weight reduction is consistent with the program’s behavioral focus and prior mindfulness-based trials, it may still be perceived as a limitation by patients and clinicians who prioritize weight outcomes. Future studies should therefore assess weight trajectories over extended follow-up durations, while continuing to evaluate mindful eating primarily as a behavioral mechanism rather than a weight-loss intervention.

Collectively, these constraints motivate the following priorities for future research. Future studies should consider stratified randomization by key demographic and clinical variables, aim to replicate these findings in more diverse samples, and incorporate longer-term follow-up to assess durability of behavioral changes. Additionally, studies should examine objective behavioral and physiological markers and conduct mediation analyses exploring the role of interoceptive awareness, emotion regulation, and self-regulation capacities to clarify mechanisms of action. The qualitative data collected in this study also offer an opportunity to further refine intervention content and assess perceived barriers and facilitators to change.

From a clinical implementation perspective, the Mind-Eat program’s structured 8-week format aligns with established group therapy models and may facilitate standardized delivery across healthcare systems. While the higher intensity represents an investment of resources, the sustained behavioral improvements at follow-up suggest potential cost-effectiveness through reduced long-term healthcare utilization. However, healthcare systems will need to weigh the program’s resource requirements against its demonstrated clinical benefits when considering implementation alongside existing patient education pathways.

## Conclusions

This randomized controlled trial provides evidence that the Mind-Eat program, a structured, mindfulness-based intervention, improves eating-related behaviors in individuals with overweight or obesity. Compared with treatment as usual, consisting of an intuitive eating–oriented TPE program, the Mind-Eat intervention led to greater reductions in emotional and external eating, alongside improvements in mindful and intuitive eating. Although no short-term effects were observed on weight, these behavioral changes highlight its potential as a complementary approach in multidisciplinary obesity care. Findings should be interpreted in light of attrition, although feasibility in a real-world outpatient setting was acceptable. Despite baseline BMI differences, results were consistent across BMI categories. Future research should replicate these findings with longer follow-up, establish clinically meaningful thresholds.

## Supplementary Information

Supplementary Material 1.

## Data Availability

Data will be made available on request.

## Abbreviations

WHO: World Health Organization
V1: Visit 1
TPE: Therapeutic patient education
SD: Standard deviations
REML: Restricted maximum likelihood
RCT: Randomized controlled trial
PSS: Perceived Stress Scale
PNNS: French National Nutrition and Health Program
MECL: Mindful eating conscious living
MEAL: Mindful eating and living
ME: Mind-Eat
MBSR: Mindfulness-based stress reduction
MBIs: Mindfulness-based interventions
MB-EAT: Mindfulness-Based Eating Awareness Training
MAR: Missing at random
LMM: Linear mixed models
IReSP: Institute for Public Health Research
IES-2: Intuitive Eating Scale-2
HAD: Hospital Anxiety and Depression Scale
FFMQ: Five Facet Mindfulness Questionnaire
DEBQ: Dutch Eating Behavior Questionnaire
BMI: Body mass index
BES: Binge Eating Scale
