# Supplementary material for: The mind-eat program leads to greater improvements in mindful, emotional, and external eating compared to intuitive eating-oriented education in adults with overweight or obesity: a randomized controlled trial
Source: Int J Behav Nutr Phys Act. 2026 May 21;23:72. doi: 10.1186/s12966-026-01931-y (PMC13377856; doi:10.1186/s12966-026-01931-y)
Supplement: Supplementary file 1 — Supplementary Material 1. [file 12966_2026_1931_MOESM1_ESM.docx]

# Supplemental Data 1. Rationale and cultural adaptations of the Mind-Eat program (French version)

The Mind-Eat program was originally adapted from the manual developed by Alberts et al. (2012). To meet the specific needs of a French clinical population with obesity, several modifications were implemented. These changes ensured cultural relevance, linguistic clarity, and feasibility in a hospital-based therapeutic patient education (TPE) context. Adaptations fell into three categories: (1) linguistic adjustments, (2) examples aligned with French eating habits, and (3) reinforcement of experiential practices.

| **Session** | **Session modifications** | **Exercises & Home practice modifications** |
| --- | --- | --- |
| 1 | Clarified definition of mindfulness and mindful eating | Body scan; mindful check-in before meals |
| 2 | Simplified 'automatic pilot' wording | Breathing space practice |
| 3 | Adapted food craving examples to French context | Raisin exercise; craving log |
| 4 | Translated self-compassion terms with culturally appropriate expressions | Self-kindness journaling |
| 5 | Clarified terminology of emotional eating | Emotion labeling practice |
| 6 | Reworded acceptance vs avoidance strategies | Urge surfing exercise |
| 7 | Adapted relapse prevention scenarios | Planning for high-risk situations |
| 8 | Harmonized gratitude expressions | Gratitude journaling; mindful eating plan |

# Supplemental Data 2. Overview of the Mind-Eat program sessions

All sessions followed a standardized structure, including: opening mindfulness practice (~10 minutes), review of home practice and group feedback, core experiential exercises, inspirational reading, and closing meditation.

| **Session** | **Theme / Content** | **Core practices** | **Home practice** |
| --- | --- | --- | --- |
| Session 1. The paradox of acceptance and change | Discovering mindfulness and eating patterns; introduction to the raisin exercise. | Opening meditation; body scan; raisin exercise; group sharing. | Daily body scan; mindful physical activity; observation of eating; form completion; setting weekly intentions; inspirational reading. |
| Session 2. Living with annoyances | Strengthening mindfulness in the face of difficulties; identifying obstacles and facilitators. | Mindful breathing; mindful eating; reflective sharing. | Mindful breathing before meals; observation of triggers; mindful eating journal; intention setting; inspirational reading. |
| Session 3. Breath and body awareness | Using breathing and movement as anchors; introduction to mindful walking and mindful movement. | Mindful walking; mindful movement; body scan; mindful eating. | Mindful breathing; movement awareness; observation of eating and movement; form completion; intention setting. |
| Session 4. Managing desires and difficult emotions | Learning to face impulses and practicing self-regulation strategies. | Breathing space; grounding techniques; mindful eating. | Mindful eating journal; trigger tracking; self-reflection on reactions; inspirational reading. |
| Session 5. Welcoming your body and the present moment | Exploring automatic behaviors and body image. | Body scan; mindful eating; ice cube exercise; image exploration. | Observation of social eating situations; mindful eating; movement reflection; values clarification; inspirational reading. |
| Session 6. Thoughts are not facts | Identifying automatic thoughts, self-criticism, and emotional regulation strategies. | Inner critic identification; breathing space; mindful eating. | Thought log; self-compassion exercises; eating observation; physical activity planning; inspirational reading. |
| Session 7. Taking care of oneself | Connecting eating behaviors with emotions and self-care. | Self-reflection; body scan; mindful eating; sharing circle. | Mindful breathing; self-care planning; intention setting; movement and eating log; inspirational reading. |
| Session 8. How to continue? | Reviewing progress, consolidating learning, preparing for autonomy. | Personal review; letting go reflection; group meditation. | Personal goals; real-life practice plan; review of tools and supports; inspirational reading. |
| Follow-up session. Integration and long-term support | Reflecting on progress and challenges post-program. | Meditation; body scan; group discussion; mindful eating. | Reflection journal; relapse strategies; future commitment plan; inspirational reading. |

**Standardized Home Practice Tools**

- **Audio recordings:** Body scan (20 min), guided meditations (10-15 min), mindful breathing (5-10 min)
- **Structured reflective journal:** Daily observations (hunger/satiety, emotions, triggers), weekly intentions, self-assessment
- **Inspirational readings:** Short texts on mindfulness, intuitive eating, and self-compassion

# Supplemental Data 3. Overview of the TPE workshops

The TPE comparator reflected the standard care pathway of the department, consistent with French national guidelines for therapeutic education. Workshops were interactive, multidisciplinary, and modular.

| **Workshop** | **Objectives** | **Facilitator** |
| --- | --- | --- |
| Shared educational assessment | Initial individual interview (~30 minutes) by phone or at the hospital. Conducted by a healthcare professional to assess participants’ needs, barriers, and expectations regarding weight management. | Nurse |
| Eating sensations | Recognize and respond to internal hunger, satiety, and fullness cues. | MD or Dietitian |
| Nutrition and health | Understand the relationship between food, health, and nutritional needs. Improve food choices. | Dietitian |
| Perception of physical activity | Distinguish between physical activity and sport. Set achievable goals and personalize activity plans. | Adapted Physical Activity Instructor |
| Sleep and health | Understand the impact of sleep on diet, weight, and overall health. Identify strategies to improve sleep quality. | MD |
| Mindful tasting | Practice tasting food mindfully, without guilt or judgment, and with full presence. | Nutritionist or Dietitian |
| Weight management follow-up group | Provide peer support and exchange on weight-related challenges. | MD |
| End-of-program assessment | Evaluate progress, unmet needs, and satisfaction with the program. | Nurse |

# Supplemental Data 4. Comparative overview of Mind-Eat vs TPE

| **Feature** | **Mind-Eat (ME)** | **TPE (control)** |
| --- | --- | --- |
| Structure | 8 fixed sessions + 1 follow-up | 3 core workshops + optional modules |
| Theoretical foundation | Mindfulness-based, experiential learning | Cognitive-educational, intuitive eating principles |
| Delivery | Single trained mindfulness instructor | Multidisciplinary team (dietitian, nurse, APA instructor, psychologist) |
| Core focus | Interoceptive awareness, non-reactivity, self-compassion | Nutrition knowledge, internal cue recognition, lifestyle advice |
| Homework | Daily meditation, mindful eating logs, gratitude journaling | Food diaries, personalized action plans |
| Duration | ~12 h total | ~4 h core (variable with optional workshops) |

**Supplemental Data 5. Description of assessment instruments**

| **Instrument** | **Construct assessed** | **Subscales / dimensions** | **Scoring** | **Interpretation** | **Psychometric properties** |
| --- | --- | --- | --- | --- | --- |
| **Mind-Eat Scale** (13) | Mindful eating | Awareness, Non-reactivity, Openness, Gratitude, Non-judgment, Hunger/Satiety (6 subscales; 4 items each) | 24 items, Likert scale (1–5); mean score computed | Higher scores indicate greater mindful eating | Validated in French population; good construct validity (CFA), internal consistency, and test–retest reliability |
| **Intuitive Eating Scale-2 (IES-2)** (14) | Adaptive eating behavior | Eating for physical rather than emotional reasons; Reliance on hunger and satiety cues; Unconditional permission to eat | Likert scale (1–5); mean score computed | Higher scores indicate greater intuitive eating | Widely validated; good internal consistency and construct validity |
| **Binge Eating Scale (BES)** (16) | Binge eating severity | Single global score | 16 items; total score | Higher scores indicate greater binge eating severity | Validated instrument; good reliability and validity |
| **Dutch Eating Behavior Questionnaire (DEBQ)** (17) | Eating behaviors | Emotional eating; External eating; Restrained eating | Likert scale; subscale scores computed | Higher scores indicate greater expression of each eating behavior | Well-validated; strong internal consistency |
| **Perceived Stress Scale (PSS)** (18) | Perceived stress | Single global score | 10 items; total score | Higher scores indicate greater perceived stress | Validated; good reliability |
| **Hospital Anxiety and Depression Scale (HAD)** (19) | Anxiety and depression | Anxiety (HAD-A); Depression (HAD-D) | 14 items; subscale scores | Higher scores indicate greater symptom severity | Widely used; good psychometric properties |
| **Five Facet Mindfulness Questionnaire (FFMQ)** (20) | Trait mindfulness | Observing, Describing, Acting with awareness, Non-judging, Non-reactivity | Likert scale; subscale scores | Higher scores indicate greater mindfulness | Validated; good reliability |
| **Ricci–Gagnon Scale** (21) | Physical activity level | Single score | Composite score | Higher scores indicate higher physical activity | Used in clinical settings; acceptable validity |
